# Current Trends and Research Hotspots in Pancreatic Stellate Cells: A Bibliometric Study

**DOI:** 10.3389/fonc.2022.896679

**Published:** 2022-06-01

**Authors:** Zhaoming Yang, Zhiqin Xie, Jian Wan, Bo Yi, Tao Xu, Xiaorong Shu, Zhijian Zhao, Caixi Tang

**Affiliations:** ^1^ Department of Hepatobiliary, Pancreatic and Splenic Surgery, Zhuzhou Hospital Affiliated to Xiangya School of Medicine, Central South University, Zhuzhou, China; ^2^ Department of Hepatobiliary Surgery, Sun Yat-sen Memorial Hospital, Sun Yat-sen University, Guangzhou, China; ^3^ Medical Records Statistics Center, Zhuzhou Hospital Affiliated to Xiangya School of Medicine, Central South University, Zhuzhou, China

**Keywords:** pancreatic stellate cells, acute/chronic pancreatitis, pancreatic cancer, bibliometric analysis, research hotspots

## Abstract

**Background:**

Pancreatic stellate cells (PSCs) play crucial roles in acute/chronic pancreatitis and pancreatic cancer. In this study, bibliometric analysis was used to quantitatively and qualitatively analyze the literature related to PSCs from 1998-2021 to summarize the current trends and research topics in this field.

**Methods:**

Relevant literature data were downloaded from the Science Citation Index Expanded Web of Science Core Collection (WoSCC) on April 07, 2021, using Clarivate Analytics. Biblioshiny R packages, VOSviewer, Citespace, BICOMB, gCLUTO, and the Online Analysis Platform of Literature Metrology (http://bibliometric.com) were used to analyze the manually selected data.

**Results:**

A total of 958 relevant studies published in 48 countries or regions were identified. The United States of America (USA) had the highest number of publications, followed by the People’s Republic of China, Germany, and Japan. Tohoku University (Japan), the University of New South Wales (Australia), the University of Texas MD Anderson Cancer Center (USA), Technical University of Munich (Germany), and University of Rostock (Germany) were the top five institutions with most publications. Nine major clusters were generated using reference co-citation analysis. Keyword burst detection revealed that progression (2016-2021), microenvironment (2016-2021), and tumor microenvironment (2017-2021) were the current frontier keywords. Biclustering analysis identified five research hotspots in the field of PSCs during 1998-2021.

**Conclusion:**

In this study, a scientometric analysis of 958 original documents related to PSCs showed that the research topics of these studies are likely in the transition from acute/chronic pancreatitis to pancreatic cancer. The current research trends regarding PSCs are related to pancreatic cancer, such as tumor microenvironment. This study summarizes five research hotspots in the field of PSCs between 1998 and 2021 and thus may provide insights for future research.

## Introduction

Pancreatic stellate cells(PSCs)are star-shaped fibroblasts and were first isolated and characterized two decades ago (1, 2). They are resident cells of the pancreas and comprise about 4-7% of the normal human pancreatic parenchyma cells (3, 4). PSCs exist in a quiescent state characterized by abundant vitamin A and albumin containing fat droplets in the cytoplasm under physiological conditions (5). Moreover, they are involved in the maintenance of pancreatic tissue architecture (6, 7). Various extracellular stimuli can activate PSCs. For instance, TGF-β1 (one of the most potent regulatory cytokines in fibrosis)can activate PSCs by inducing JNK and ERK over-expression (8). Reactive oxygen species can induce activation, invasion, migration, and glycolysis of PSCs (9). Chronic hyperglycemia can activate PSCs and promote the interaction between PSCs and pancreatic cancer cells (10). Activated PSCs play a crucial role in the development of pathological pancreatic fibrosis (11, 12), which characterizes chronic pancreatitis (13, 14) and pancreatic cancer-associated desmoplastic reaction (7, 15). Therefore, chronic pancreatitis and pancreatic cancer can be treated using anti-fibrotic agents, such as vitamin analogs, targeting PSCs (16). Nevertheless, the PSCs-targeting strategy requires further analysis for its clinical application. Besides, the exact role of PSCs in chronic pancreatitis and pancreatic cancer should be further elucidated. There have been plummeting publications related to PSCs worldwide over the past two decades. Therefore, it is necessary to conduct a comprehensive analysis of the progress and development trends in the field of PSCs.

Bibliometric analysis has been used to quantitatively and qualitatively analyze specific literature based on mathematics and statistics. It can systematically visualize the development of the research topics and show the current trends and hotspots of a certain field. Moreover, it has been widely used across many research fields (17). Citespace software (18), VOSviewer (visualizations of similarities, van Eck and Waltman, Leiden University, Leiden, Netherlands) (19) software, and the Online Analysis Platform of Literature Metrology (http://bibliometric.com/) can be used for detailed analysis of countries/regions, institutions, journals, authors, co-citations and keywords of a certain field. BICOMB and gCLUTO software can be used to determine the relationship between frequent keywords and source documents (20, 21).

Several review studies have revealed that PSCs play key roles in acute/chronic pancreatitis and pancreatic cancer. However, there is no systematic bibliometric analysis that can describe the research trends and hotspots of PSCs and adequately characterize the overall framework of PSCs analysis. Visualization methods can help researchers intuitively understand the main points. As a result, a combination of bibliometric and visualization review, known as two-level research (22) has been widely applied to bridge this gap. This study aimed to systematically analyze the developments in the research field of PSCs and provide a comprehensive understanding and an intuitive visualization of the current trends and research topics for future research.

## Methods

Literature search was conducted on April 07, 2022, and all documents were obtained from the Science Citation Index Expanded Web of Science Core Collection (WoSCC) using Clarivate Analytics. Two independent authors (Zhaoming Yang and Xiaorong Shu) verified the front page to exclude irrelevant publications. The following search keywords were used: “Pancreatic stellate cell*” OR “Cell*, Pancreatic stellate” OR “Stellate cell*, Pancreatic” OR “Pancreatic stellate shaped cell*” OR “Pancreatic periacinar stellate cell*”. Notably, the topic (*TS*) module of WoSCC includes the paper title (*TI*), abstract (*AB*), author keywords (*DE*) and keywords plus (*ID*). Keywords plus are words or phrases that frequently appear in the titles of an article’s references, but do not appear in the title of the article. Therefore, these search keywords were searched using the title (*TI*), abstract (*AB*), and author keywords (*DE*) in advanced search as previously described to obtain more accurate bibliometric results (23, 24). Only papers published from 1998-01-01 to 2021-12-31 and in English were included in this analysis. Subsequently, only articles or review articles were included. Other types of publications, including meeting abstracts, proceeding papers, editorial materials, letters, corrections, book chapters, retracted publications, and early access, were excluded.

### Data Analysis

First, data were downloaded from the Science Citation Index Expanded Web of Science Core Collection using Clarivate Analytics. Full records and cited references were saved in plain text format. A polynomial regression model was constructed using Microsoft Office Excel 2016 (Microsoft, Redmond, Washington, USA) to predict the annual growth trend of publications. The Citespace software V5.8.R3 was then used for references co-citation analysis, and keywords burst detection using the following setting: Time slicing from January 1998 to December 2021, years per slice; 1. The selection criteria for the analysis of references co-citation was set as g-index (k=25). The VOSviewer software (1.61.17) was used for collaboration network analysis between countries, institutions, authors and keywords. For keywords analysis, author keywords and keywords plus units were analyzed. The minimum occurrence number of a keyword was chosen as 5 for the construction of collaboration networks. Meanwhile, all documents were downloaded in the UTF-8 format and imported into the Online Analysis Platform of Bibliometrics (http://bibliometric.com/). The annual publications of the top ten countries or regions were constructed, and “partnership analysis” was chosen for intercountry analysis. Finally, a binary matrix of keywords and source documents was constructed using the BICOMB software. The semantic relationship between keywords and source documents was demonstrated based on mountain and matrix visualization *via* gCLUTO software.

## Results

### Current Publication Trends of Pancreatic Stellate Cells

**Figure 1:**
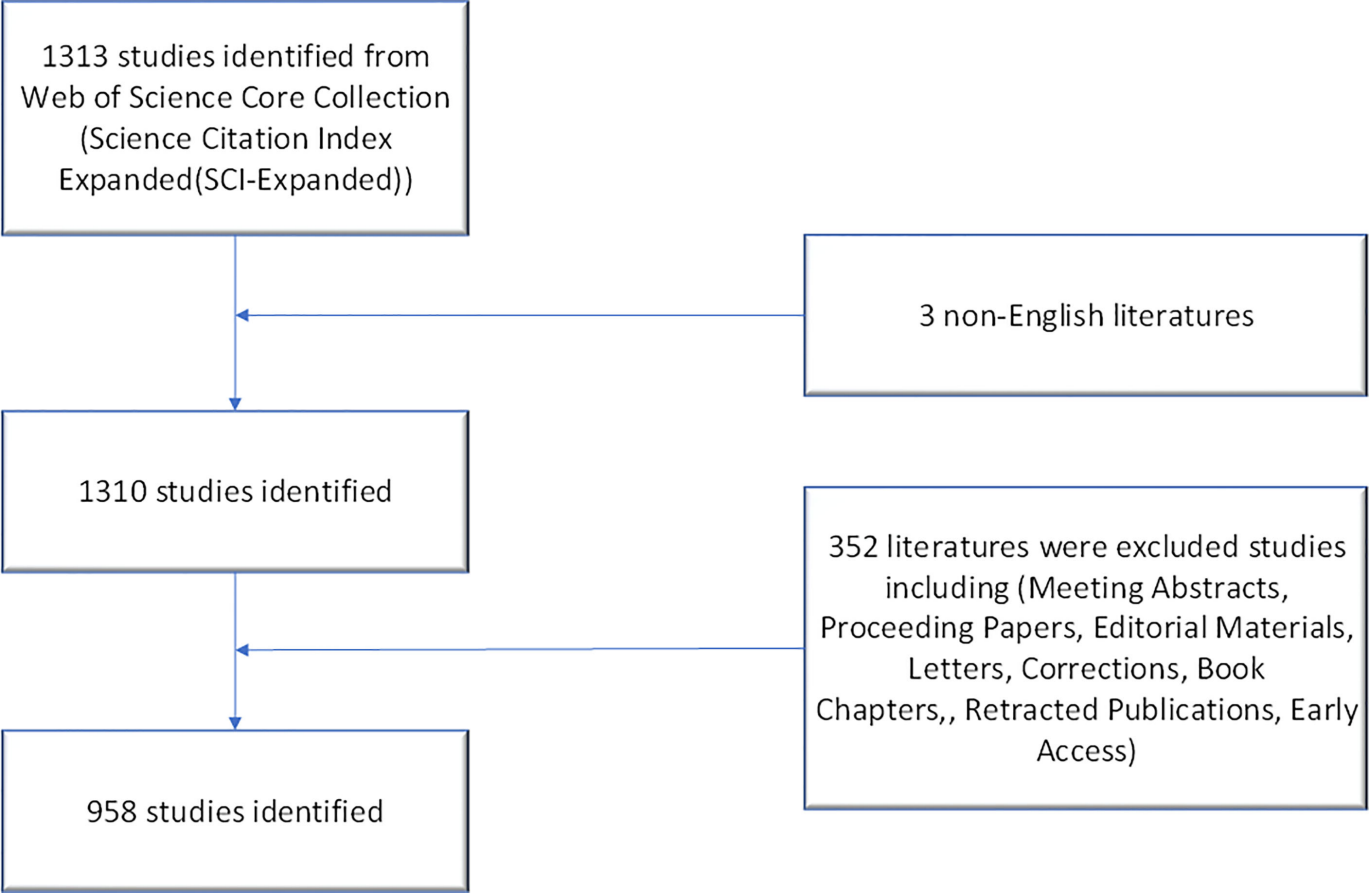
Flow diagram of the search strategy and protocol of the study.

**Figure 2:**
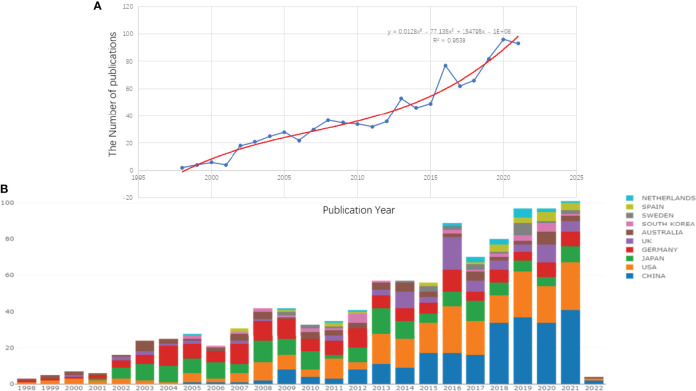
Growth trends of publications on PSCs from 1998 to 2021. The polynomial regression fitting curve of annual publications **(A)** and the number of annual publications of the top 10 countries/regions **(B)**. This analysis was conducted using the Online Analysis Platform of Literature Metrology (http://biblimetric.com). The bar chart reflects the number of online articles online per year.

Clarivate Analytics showed that 958 papers related to pancreatic stellate cells were published from 1998 to 2021 and recorded in the Science Citation Index Expanded Web of Science Core Collection (**Figure 1**). The chronological distribution of PSCs-related publications is shown in **Figure 2A**. The number of annual publications steadily increased during the last two decades and peaked in 2020. There were only two publications in 1998, which increased to 96 in 2020, implying that the field of pancreatic stellate cells attracted increasing attention. Original articles accounted for 85.6%, and reviews accounted for 14.4%. A polynomial regression model was constructed to predict the number of annual publications (*R* ^2 =^ 0.9539) (**Figure 2A**). The prediction showed that there would be about 148 publications in 2025 based on the fitting curve. The number of publications online each year, including articles published in 2022, is shown in **Figure 2B** (drawn using the Online Analysis Platform of Bibliometrics (http://bibliometric.com/).

### Active Countries and Institutions in the Field of Pancreatic Stellate Cells

**Figure 3:**
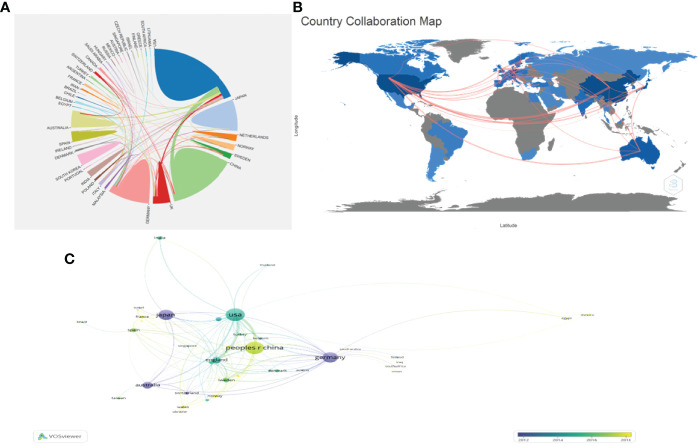
Collaboration networks between countries/regions in the field of PSCs from 1998 to 2021. The collaboration networks between countries/regions conducted using Biblioshiny packages **(A)**, the Online Analysis Platform of Literature Metrology (http://biblimetric.com) **(B)**, and the VOSviewer software **(C)**.

The number of annual publications for the top 10 countries is shown in **Figure 2B**. The United States of America published the most papers, followed by the People’s Republic of China, Germany, and Japan. However, People’s Republic of China had more annual publications than the USA since 2018, indicating that the research field of PSCs attracted more scholars in People’s Republic of China (**Figure 2B**). In this study, the collaboration networks between countries were constructed using the VOSviewer software (**Figure 3C**) and Biblioshiny package (**Figure 3B**). The analysis showed that at least 48 countries or regions have been doing research in the field of PSCs. The “partnership analysis” of the Online Analysis Platform of Literature Metrology was used for intercountry analysis. The partnership between different countries in the field of PSCs is shown in **Figure 3A**.

**Figure 4:**
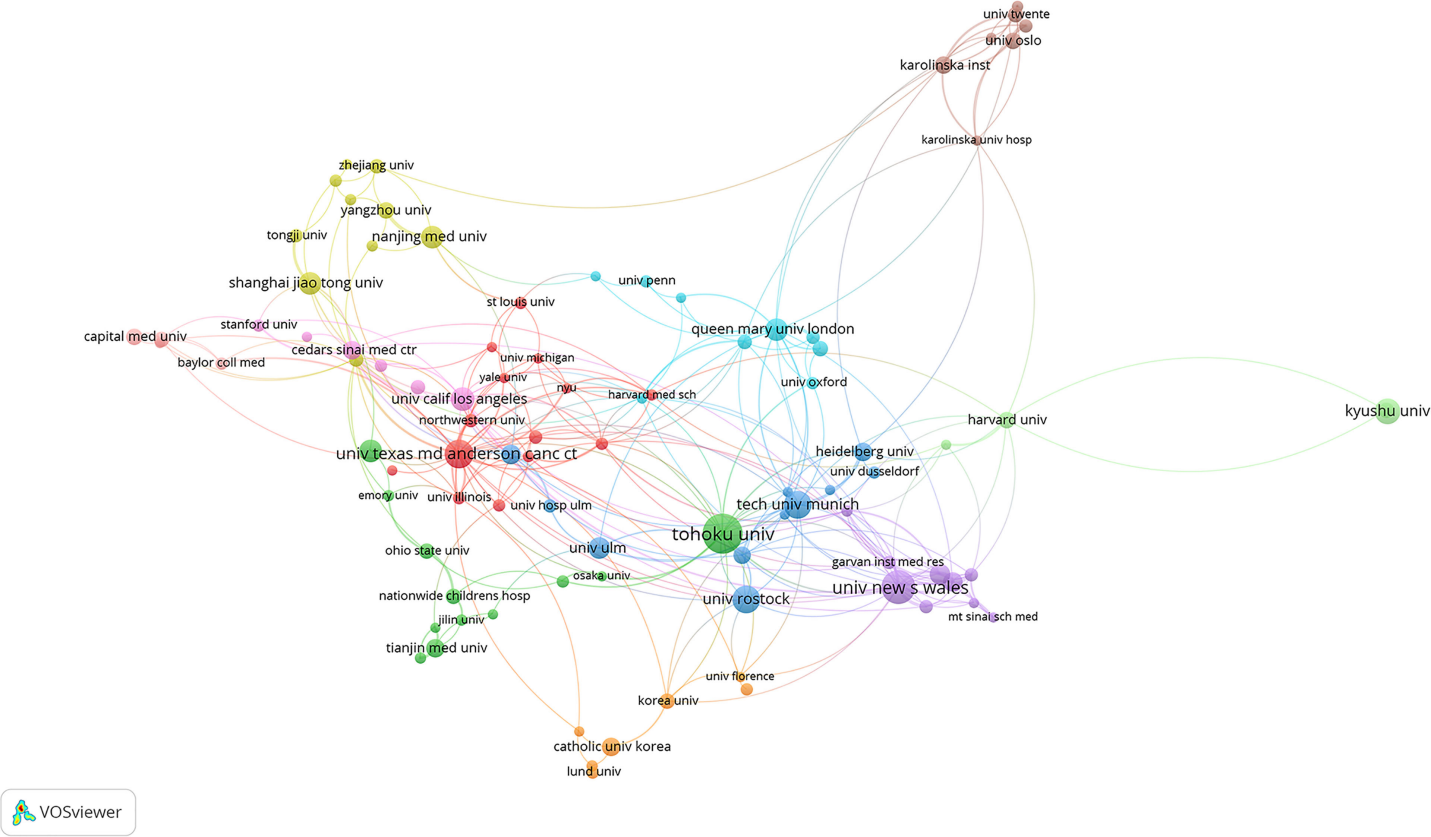
Collaboration networks between institutions in the field of PSCs from 1998 to 2021. This analysis was conducted using the VOSviewer software.

Tohuku University in Japan published most literatures (65). Tohuku University (Japan), University of New South Wales (Australia), the University of Texas MD Anderson Cancer Center (USA), Technical University of Munich (Germany), University of Rostock (Germany), Kyushu University (Japan), University of California Los Angeles (USA), the Xi’An Jiao Tong University (China), Queen Mary University London (UK) and Shanghai Jiao Tong University (China) were the top 10 most productive institutions. The collaboration networks among the institutions are shown in **Figure 4**.

### Productive Journals in the Field of Pancreatic Stellate Cells

**Figure 5:**
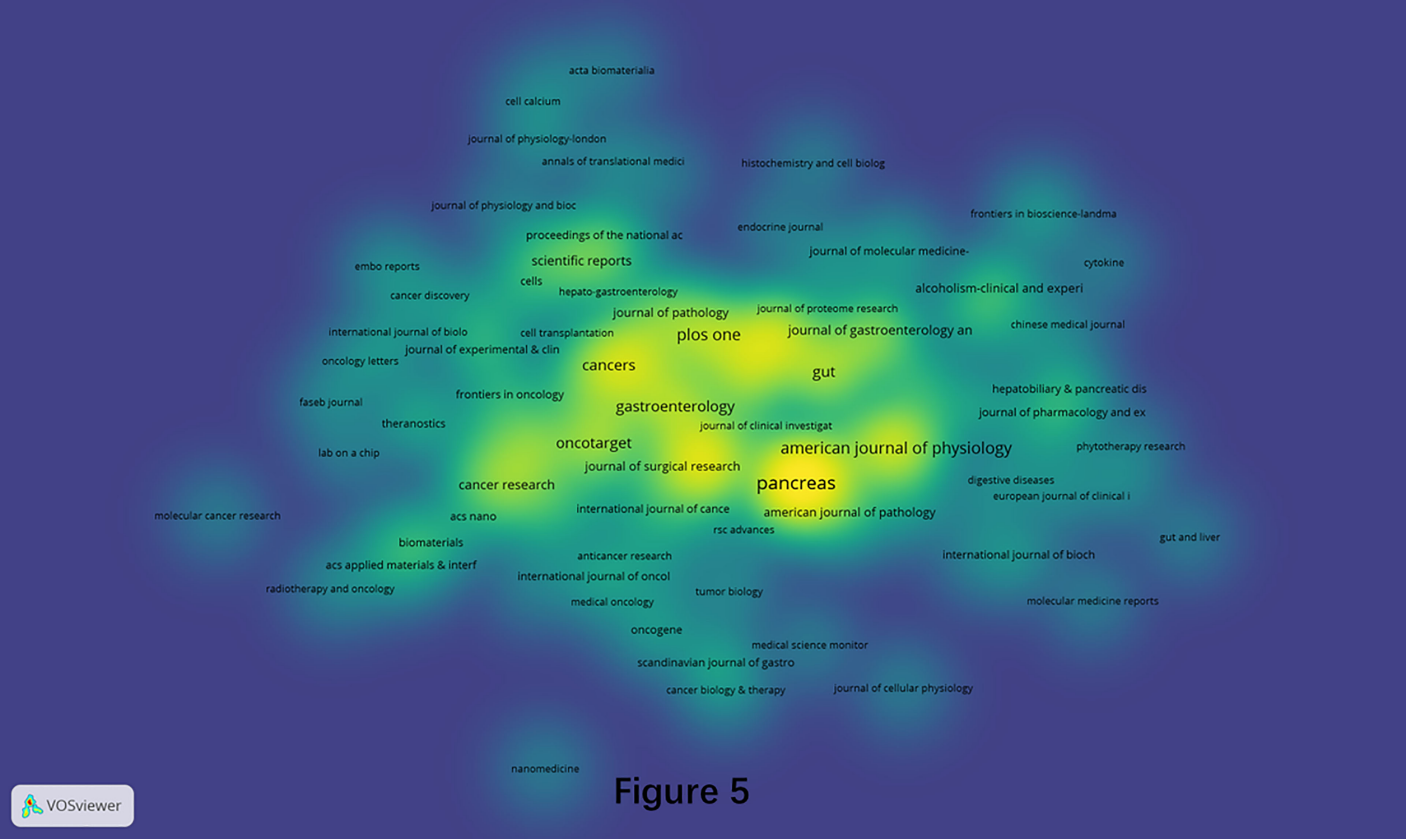
Density visualization of journals that published PSCs articles from 1998 to 2021. This analysis was conducted using the VOSviewer software.

A total of 315 journals had published pancreatic stellate cells-related papers in the current analyzed data over the last two decades (1998-2021). The density visualization constructed by the VOSviewer software is shown in **Figure 5**. The top 10 most productive and top 10 most influential journals are listed in **Table 1**. “Pancreas” ranked first with 55 papers, while “Gastroenterology” had most citations (3696).

**Table 1:** Top productive and cited journals.

No.	Journals	Count(N)	No.	Journals	Total citations
1	Pancreas	55	1	Gastroenterology	3696
2	Pancreatology	38	2	Gut	3058
3	American Journal of physiology- gastrointestinaland liver physiology	28	3	Cancer Research	2076
4	Plos One	25	4	Pancreas	1696
5	Gut	23	5	American Journal of physiology- gastrointestinal and liver physiology	1186
6	Cancers	23	6	American Journal of Pathology	1091
7	Gastroenterology	22	7	Pancreatology	958
8	Biochemicaland Biophysical Research Communications	22	8	Clinical Cancer Research	816
9	Oncotarget	21	9	Biochemicaland Biophysical Research Communications	809
10	World Journal of Gastroenterology	20	10	Laboratory Investigation	785

### Influential Authors in the Field of Pancreatic Stellate Cells

**Figure 6:**
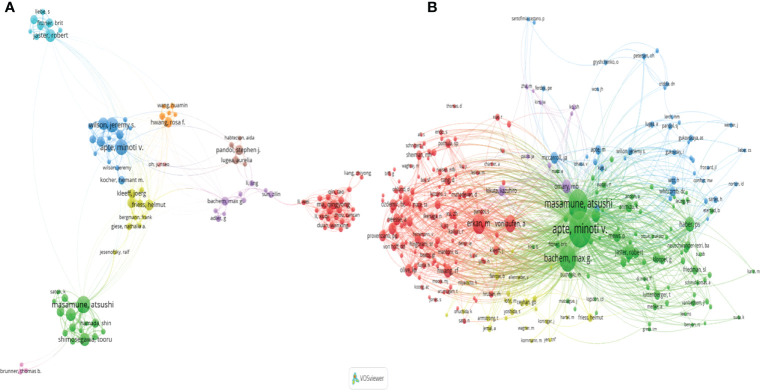
A network map showing authors **(A)** and co-cited authors involved in the field of PSCs from 1998 to 2021 **(B)**. This analysis was conducted using the VOSviewer software.

The collaboration analysis of authors conducted by the VOSviewer software is presented in **Figure 6A**. A total of 181 of 4496 authors had published at least five papers in the field of PSCs. The top 10 most productive and top 10 most co-cited authors are listed in **Table 2**. Atsushi Masamune was the most productive author with 58 published documents, while Minoti V. Apte was the most cited author with 5868 citations. Meanwhile, the collaboration networks of co-cited authors were also constructed using the VOSviewer software. Apte Minoti V. was the most co-cited author with 1371 citations, followed by Masamune Atsushi and Bachem Max G (**Figure 6B** and **Table 2**).

**Table 2:** Top productive and co-cited authors.

No.	Author	Count(N)	Co-cited Author	Citations
1	Masamune Atsushi	58	Apte,Minoti V.	1371
2	Apte,Minoti V.	48	Masamune Atsushi	977
3	Wilson,Jeremy S.	44	Bachem, Max G.	728
4	Shimosegawa, Tooru	34	Erkan, Mert	428
5	Jaster, Robert	32	Vonlaufen, A	315
6	Kikuta, Kazuhiro	31	Harber, PS	236
7	Pirola, Romano C.	31	Jaster Robert	231
8	Friess, Helmut	24	Omary, MB	203
9	Erkan, Mert	22	Hwang, RF	197
10	Xu, Zhihong	21	Phillips, Phoebe a.	197

### Popular References in the Field of Pancreatic Stellate Cells

**Figure 7:**
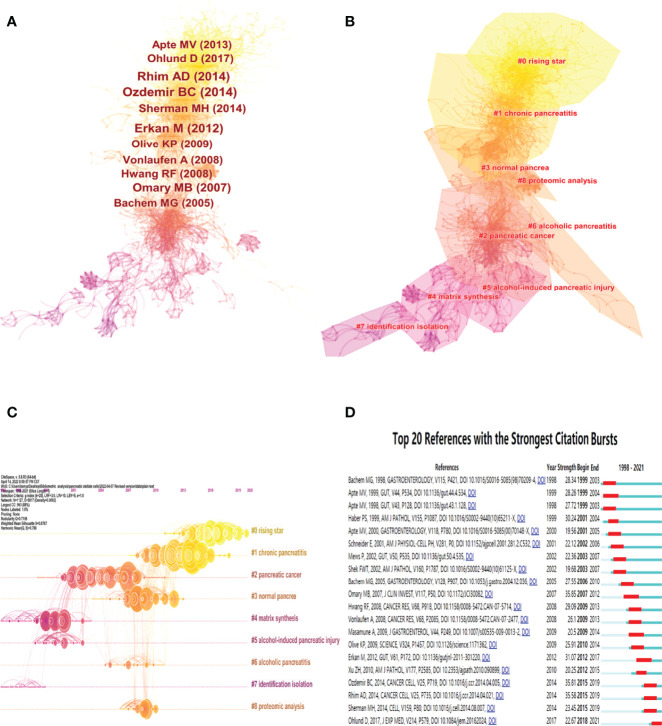
Co-citation map and cluster networks of references in the field of PSCs from 1998 to 2021. Co-citation map of 27636 valid references in the field of PSCs, with the 10 most cited references marked **(A)**. Cluster networks **(B)** and timeline view **(C)** of references in the field of PSCs. The top 20 references with the strongest citation bursts **(D)** on PSCs research. All these analyses were conducted using the Citespace software.

The included original documents and their 27636 valid distinct references were analyzed using Citespace software to assay the scientific relevance of the related publications. A frequently cited document is of great popularity and influence in its research field (25). A network of co-cited references was constructed using the following cluster setting parameters: #Years Per Slice=1, selection Criteria: g-index (k=25), pruning=None (**Figure 7**). The co-cited reference network is shown in **Figure 7A**, where the top 10 most cited references are marked. The nine major clusters of the aforementioned documents and their references generated by Citespace software are shown in **Figures 7B, C**. The Modularity Q score was 0.7156, >0.5, showing that the network was reasonably divided into loosely coupled clusters. The Weighted Mean silhouette score was 0.8767, >0.5, indicating that the clusters had acceptable homogeneity. The nine distinct clusters were marked as “#0 rising star”, “#1 chronic pancreatitis”, “#2 pancreatic cancer”, “#3 normal pancrea”, “#4 matrix synthesis”, “#5 alcohol-induced pancreatic injury”, “#6 alcoholic pancreatitis”, “#7 identification isolation” and “#8 proteomic analysis” Moreover, the top 20 references with the strongest citation bursts are shown in **Figure 7D**, indicating their significance in the field of PSCs.

### Research Topic Development of Pancreatic Stellate Cells

**Figure 8:**
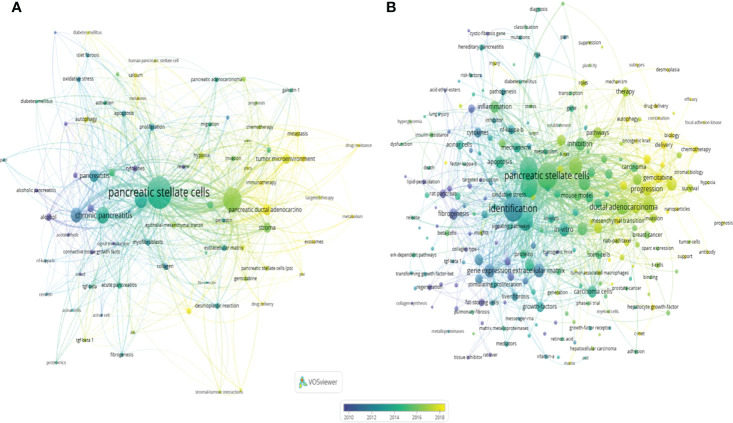
Co-occurrence of author keywords (*DE*) and keywords plus (*ID*) in the field of PSCs from 1998 to 2021. A network map of author keywords **(A)** and keywords plus **(B)** conducted by the VOSviewer software.

**Figure 9:**
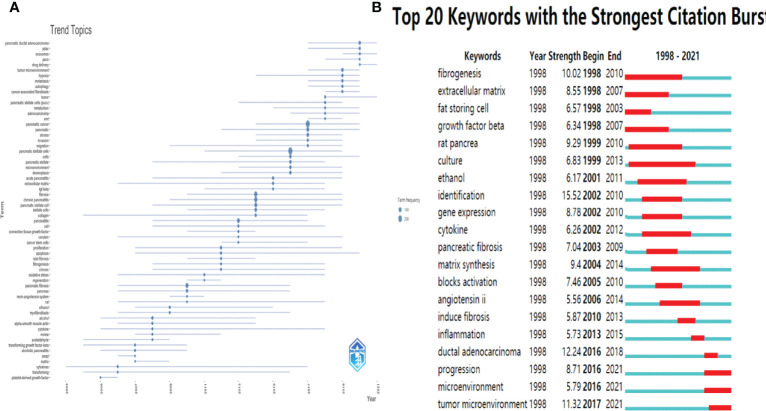
Trend Topics and Keywords burst detection in the field of PSCs from 1998 to 2021. Trend Topics **(A)** conducted by Biblioshiny packages. The top 20 keywords with strongest citation bursts **(B)** conducted by the Citespace software.

The keywords in the downloaded documents were obtained and analyzed using VOSviewer software. The “Authorkeywords” and “Keywords Plus” units of “Co-occurrence” analysis were chosen for keywords analysis, with a threshold minimum of five occurrences. A thesaurus file was applied in this analysis process to avoid the dilemma of the existence of synonyms. The results showed that 83 author keywords and 247 keywords plus met the threshold (**Figure 8**). The top three keywords with the most occurrences were pancreatic stellate cells (440 occurrences), pancreatic cancer (243 occurrences) and chronic pancreatitis (117 occurrences) for author keywords, and pancreatic stellate cells (256 occurrences), identification (236 occurrences), and fibrosis (205 occurrences) for keywords plus. The pancreatic cancer-related author keywords, such as tumor microenvironment, chemotherapy, targeted therapy, and epithelial-mesenchymal transition, occurred recently, indicating that the current hotspots or frontiers in the field of PSCs were pancreatic cancer-related (**Figure 8A**). Similarly, keywords plus, including chemotherapy, progression, drug delivery, and gemcitabine, occurred recently (**Figure 8B**). Next, trend topics analysis was performed using Biblioshiny package, and results showed that the earlier most frequent topics were alcoholic pancreatitis, pancreatic fibrosis and chronic pancreatitis, while the most recent frequent topics were cancer-associated fibroblasts, tumor microenvironment and PDAC (**Figure 9A**). Moreover, keywords burst detection was also conducted using the Citespace software (**Figure 9B**). The results showed that progression (2016-2021), microenvironment (2016-2021), and tumor microenvironment (2017-2021) were the latest strongest citation bursts.

### Research Hotspots of Pancreatic Stellate Cells

**Figure 10:**
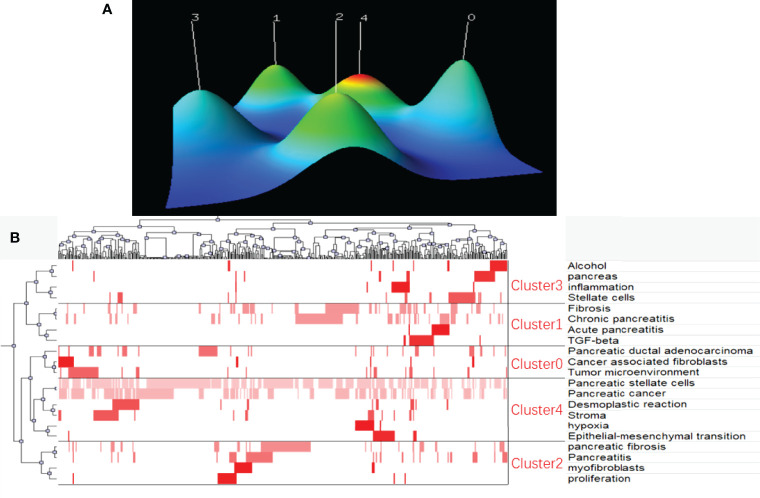
Biclustering of extremely frequent keywords and their source documents in the field of PSCs from 1998 to 2021. Mountain visualization **(A)** and matrix visualization **(B)** conducted by gCLUTO software.

Finally, keywords were extracted from original documents using BICOMB software for the analysis of research hotspots in the field of PSCs. Twenty-one keywords that appeared more than 19 times were identified and defined as extremely frequent keywords using H-index evaluation. The identified keywords accounted for 38.29% of all the keywords occurrences. The selected keywords and their source documents were saved in matrix format and subsequently analyzed using the gCLUTO software. Five different clusters were sorted using the biclustering method, and the results were visualized *via* mountain and matrix visualization. Five peaks were detected in mountain visualization, representing the five sorted clusters (**Figure 10A**). The distance between different peaks indicated the relative similarity between different clusters, while the altitude of the individual peak indicated the internal similarity of the cluster. The size and color of the peak represented the number of keywords contained and the standard deviation of the cluster, respectively. The matrix visualization of the five sorted clusters is shown in **Figure 10**. Finally, five hotspots in the field of PSCs were summarized and identified: (0) cancer-associated fibroblasts, tumor microenvironment, and PDAC; (1) TGF-beta, fibrosis, and Acute/Chronic pancreatitis; (2) myofibroblasts, pancreatic fibrosis and pancreatitis; (3) Alcohol, inflammation and pancreatic stellate cells; (4) Influence of PSCs on desmoplastic reaction, EMT, hypoxia, and stroma of pancreatic cancer.

## Discussion

In the present study, a bibliometric analysis of literature in the field of PSCs over the past two decades was conducted. The results showed that the number of PSCs-related publications steadily increased. The USA published most PSCs-related papers and had the most citations. China was second with the sixth most citations, indicating that the quality of research needs to be improved with the increasing number of publications. Germany and Australia, the two countries that first identified PSCs, were third and fifth, respectively, with the second and third most citations, respectively, indicating that the two countries had a great influence in the field of PSCs. For institutions, Tohoku university from Japan published most articles related to PSCs. The institution mainly illustrated the role of PSCs in the carcinogenesis and progression of pancreatic cancer and to find novel therapeutic targets. For instance, Tanaka Yu et al. identified that Nrf2 promotes PSC activation and Nrf2 deletion in PDCs, resulting in attenuation of the cancer-promoting role (26). Yoshida Naoki et al. also found that peritumoral stromal expression of kindlin-2, a focal adhesion protein regulating the activation of integrins, is associated with shorter recurrence-free survival after R0 resection of PDAC, indicating that kindlin-2 in PSCs promotes the progression of pancreatic cancer (27). Tohoku University researchers found that phytochemicals, such as curcumin (28) and green tea polyphenol epigallocatechin-3-gallate (29) can block PSCs activation. University of New South Wales from Australia, where Professor Minoti V Apte works, was the second most productive institution. Besides identifying pancreatic stellate cells (1) Minoti V Apte et al. also demonstrated the cell biology of PSCs and explored the role of PSCs in alcohol-induced pancreatitis (30) chronic pancreatitis (31) and pancreatic cancer (15). They recently found that the inhibition of hepatocyte growth factor (HGF)/c-MET pathway, which is upregulated in PC and mediates the interaction between cancer cells and stromal PSCs, can limit primary tumor growth and eliminate metastasis (32, 33). The University of Texas MD Anderson Cancer Center was the third most productive institution. Professor Jason B Fleming and his group were particularly interested in an extracellular matrix proteoglycan overexpressed by PSCs called lumican. They reported that lumican can directly restrain PDAC growth (34) and augment cytotoxicity of chemotherapy in PDAC (35). Xi’an Jiaotong University was the most productive institution in China and the 8th most productive institution worldwide. The researchers from Xi’an Jiaotong University were interested in the mechanisms of PSCs activation and therapeutic strategies to block its activation. For instance, Li et al. found that sonic hedgehog (SHH) is overexpressed in pancreatic cancer tissues They also showed that stromal PSCs activated by SHH paracrine signaling promotes perineural invasion in pancreatic cancer (36). Zhang et al. showed that Yap-Myc signaling, involved in pancreatic cancer metabolism, induces pancreatic stellate cell activation by regulating glutaminolysis (37). Moreover, inhibition of PSCs activation by resveratrol (38) or inhibiting heat shock protein-90 (39) can effectively ameliorate the malignant progression or enhance the efficacy of PD-1 blockade therapy of pancreatic ductal adenocarcinoma.

The timeline view of the references and keyword burst detection, and the keyword occurrence analysis indicate research trends in the field of PSCs. PSCs have been identified for over 20 years. Moreover, various researchers worldwide have reported the functions of these cells in pancreatitis and pancreatic cancer. For instance, toxic factors, such as ethanol, FA or bile, induces a sustained Ca^2+^ signal and nitric oxide (NO) formation in PSCs, and NO diffusion, thus damaging pancreatic acinar cells (PAC) and increasing the amount of trypsin in acute pancreatitis. The elevated trypsin level further stimulates NO production in PSCs and triggers the necrotic amplification loop in acute pancreatitis (40, 41). In chronic pancreatitis, exogenous stimuli activate PSCs, leading to the production of pathological fibrosis (3). Therefore, targeting PSCs is a potential therapeutic strategy for chronic pancreatitis. Moreover, retinoic acid (RA) significantly induces PSCs apoptosis, inhibits PSCs proliferation, and suppresses ECM production of PSCs, thus ameliorating chronic pancreatitis (42). However, this should be further validated in clinical trials.

Keywords Burst detection showed that the tumor microenvironment is one of the research frontiers in the field of PSCs. PSCs and their secreted extracellular matrix proteins are key components of pancreatic cancer tumor microenvironment. PSCs act as a protumor factor. Particularly, PSCs promote the proliferation, invasion, angiogenesis, immune-escape, and chemo-resistance of pancreatic ductal adenocarcinoma. Activated PSCs secret elevated levels of cytokines and growth factors, such as IL-6 (43) and vascular endothelial growth factor (44). These secreted factors promote the proliferation, invasion, and angiogenesis of cancer cells (45). Meanwhile, exosomal microRNAs derived from PSCs can promote pancreatic cancer. For instance, Li et al. reported that PSCs-derived exosomal miR-5703 promotes the proliferation of pancreatic cancer cells by downregulating the tumor suppressor gene CMTM4 (46). Cao et al. also reported that exosomal microRNAs derived from hypoxic PSCs promote proliferation and invasion of pancreatic cancer by downregulating PTEN and activating AKT signaling (47). A research group from Tohoku University assessed the microRNA expression profile in PSC-derived exosomes and found that PSCs-derived exosomes can stimulate the proliferation, migration, and chemokine gene expression in pancreatic cancer cells (48). Besides, activated PSCs promote the expansion and differentiation of immunosuppressive MDSCs (49, 50) and transform macrophages into pro-tumoral M2 macrophages (51). Activated PSCs sequester CD8+ T cells to reduce their migration and infiltration of the juxtatumoral compartment of PDAC (52). Moreover, activated PSCs-secreted galectin-1 promotes the apoptosis of CD3+CD4+ and CD3+CD8+ effector T cells, developing a PSC-dependent immunoprivilege in the pancreatic cancer microenvironment (53). Activated PSCs also produce abundant ECM proteins and induce a desmoplastic reaction, leading to intratumoral hypoxia and chemoresistance (54). The PSCs-secreted ECM molecules, including collagens, fibronectin, and laminin, are densely deposited in the pancreatic cancer microenvironment, forming a physical barrier for drug delivery (55, 56).

As a result, several strategies have been explored to modulate PSCs activation. Particularly, a phase 1b clinical trial has validated that all-trans-retinoic-acid (ATRA) is safe and tolerable (57). ATRA, an active metabolite of vitamin A, inhibits PSCs activation (58) restores mechanical quiescence in PSCs (59) and suppresses extracellular matrix remodeling, thus inhibiting local cancer cell invasion (60). Enzymatic targeting of hyaluronan, a stromal component, can significantly reduce the number of activated PSCs, decrease tumor volumes and increase overall survival in KPC mice (61, 62). Some studies also focused on selective phytochemicals targeting pancreatic stellate cells as new anti-fibrotic agents for chronic pancreatitis and pancreatic cancer (16). Phytochemicals, such as curcumin (28) rhein (63, 64) and green tea catechin derivatives (29, 65) can inhibit PSCs activation.

However, this study has some limitations. First, this study only used data from the SCI-Expanded WoSCC, which could not reflect all the researches in the field of PSCs. Second, this study only assessed English articles or reviews, ignoring significant studies in other languages. Finally, this study did not include the most recent publications in 2022, which represents the frontiers in the field of PSCs.

In conclusion, the present study provided the trends of PSCs based a systematic bibliometric analysis and visualization review. Therefore, it bridges the gap and identifies the hotspots and frontier topics in the field of PSCs, providing a basis and guidance for future study. The United States of America was the most productive country, followed by People’s Republic of China, Germany, Japan, and Australia. Tohoku University from Japan was the most productive institution, while the University of New South Wales from Australia had the most citations. The most popular and influential journals were “Pancreas”, and “Gastroenterology”, respectively. Atsushi Masamune and Minoti V. Apte were the most productive and the most-cited authors, respectively. The research hotspots included: (1) TGF-beta, fibrosis, and Acute/Chronic pancreatitis; (2) myofibroblasts, pancreatic fibrosis, and pancreatitis; (3) alcohol, inflammation, and pancreatic stellate cells; (4) Influence of PSCs on desmoplastic reaction, EMT, hypoxia, and stroma of pancreatic cancer. The research frontier was “tumor microenvironment”.

## Data Availability Statement

The original contributions presented in the study are included in the article/supplementary material. Further inquiries can be directed to the corresponding authors.

## Author Contributions

ZY, ZZ, ZX and CT designed this study. ZY and XS performed the search and collected data. JW, BY and TX re-checked data. ZY and XS performed analysis. All authors contributed to the article and approved the submitted version.

## Conflict of Interest

The authors declare that the research was conducted in the absence of any commercial or financial relationships that could be construed as a potential conflict of interest.

## Publisher’s Note

All claims expressed in this article are solely those of the authors and do not necessarily represent those of their affiliated organizations, or those of the publisher, the editors and the reviewers. Any product that may be evaluated in this article, or claim that may be made by its manufacturer, is not guaranteed or endorsed by the publisher.
